# Delay-dependent impairment of spatial working memory with inhibition of NR2B-containing NMDA receptors in hippocampal CA1 region of rats

**DOI:** 10.1186/1756-6606-6-13

**Published:** 2013-03-13

**Authors:** Xue-Han Zhang, Shu-Su Liu, Feng Yi, Min Zhuo, Bao-Ming Li

**Affiliations:** 1 Institute of Neurobiology, and State Key Laboratory of Medical Neurobiology, Institutes of Brain Science, Fudan University, 138 Yi Xue Yuan Road, Shanghai, 200032, China; 2 Center for Neuropsychiatric Diseases, Institute of Life Science, Nanchang University, Nanchang, 330031, China; 3 Department of Physiology, Faculty of Medicine, University of Toronto, 1 King’s College Circle, Toronto, ON, M5S 1A8, Canada

**Keywords:** NR2B, Hippocampus, Working memory, T-maze, Rat

## Abstract

Hippocampal N-methyl-D-aspartate receptor (NMDAR) is required for spatial working memory. Although evidence from genetic manipulation mice suggests an important role of hippocampal NMDAR NR2B subunits (NR2B-NMDARs) in spatial working memory, it remains unclear whether or not the requirement of hippocampal NR2B-NMDARs for spatial working memory depends on the time of spatial information maintained. Here, we investigate the contribution of hippocampal NR2B-NMDARs to spatial working memory on delayed alternation task in T-maze (DAT task) and delayed matched-to-place task in water maze (DMP task). Our data show that infusions of the NR2B-NMDAR selective antagonists, Ro25-6981 or ifenprodil, directly into the CA1 region, impair spatial working memory in DAT task with 30-s delay (not 5-s delay), but severely impair error-correction capability in both 5-s and 30-s delay task. Furthermore, intra-CA1 inhibition of NR2B-NMDARs impairs spatial working memory in DMP task with 10-min delay (not 30-s delay). Our results suggest that hippocampal NR2B-NMDARs are required for spatial working memory in long-delay task, whereas spare for spatial working memory in short-delay task. We conclude that the requirement of NR2B-NMDARs for spatial working memory is delay-dependent in the CA1 region.

## Introduction

Spatial working memory is a dynamic encoding process and active representation of spatial information over a short time, through which spatial information is acquired and is updated repeatedly owing to continuously changing spatial information (Olton, 1979). The contents of spatial working memory can represent a recently visited place that is temporarily held in mind to guide forthcoming behaviour [1,2]. The capacity of spatial working memory has been examined using tasks with a delay, such as the delayed alternation task and delayed matching-to-place task [3]. The hippocampus is an essential structure for spatial working memory. Inactivation of or lesion to the CA1 region produces a severe deficit in the spatial working memory [4-7].

N-methyl-D-aspartate receptor (NMDAR) is a heteromer [8,9], consisting of NR1 subunits [10] and various NR2 subunits (A-D) [11]. NR2A and NR2B are the major NR2 subunits in adult forebrain. The roles of NR2A- and NR2B-NMDARs in the hippocampus-dependent spatial memory and fear memory are well established. Mice lacking the NR2A subunits exhibit a impairment in spatial memory [12]. Genetic over-expression of NR2B subunits in the adult mouse enhances both spatial memory and fear memory [13]. Pharmacological inhibition of NR2A- or NR2B-NMDARs impairs fear memory in many brain regions, including hippocampal CA1 region [14-16]. Specifically, the requirement of NR2B-NMDARs in fear memory process depends on the conditioning strength in hippocampus and other brain area [15-17], whereas the requirement of NR2A-NMDARs independs on conditioning strength [15,16].

Hippocampal NMDAR has been implicated in spatial working memory. Previous studies using non-selective NMDAR antagonists demonstrate that NMDAR are essential for spatial working memory in the CA1 region [7,18-21]. Recently, genetically modified mice demonstrate the important roles of NR2A and NR2B subunits in spatial working memory. NR2A-NMDAR knockout mice display a severe deficit in spatial working memory [22]. The mice with hippocampal NR2B-NMDAR ablation show a spatial working memory deficit for recently visited places [23]. However, it is still unclear whether the requirement of hippocampal NR2B-NMDARs for spatial working memory depends on the time of spatial information kept.

In the present study, we investigated the impact of pharmacological inhibition of CA1 NR2B-NMDARs on delayed alternation T-maze task ( a task of delayed non-matched-to-place) and delayed match-to-place water maze task, these two tasks of spatial working memory that have been widely used for spatial working memory study in rodents. In these tasks, the rat is typically cued (via a spatial stimulus) to make a choice response to obtain a food reward or to locate escape platform, but is prevented from responding until after some delay period has been imposed. The delay period determines the capacity of spatial working memory.

## Results

### Intra-CA1 inhibition of NR2B- NMDARs impairs spatial working memory in delayed alteration T-maze task with 30-s but not 5-s delay

**Figure 1:**
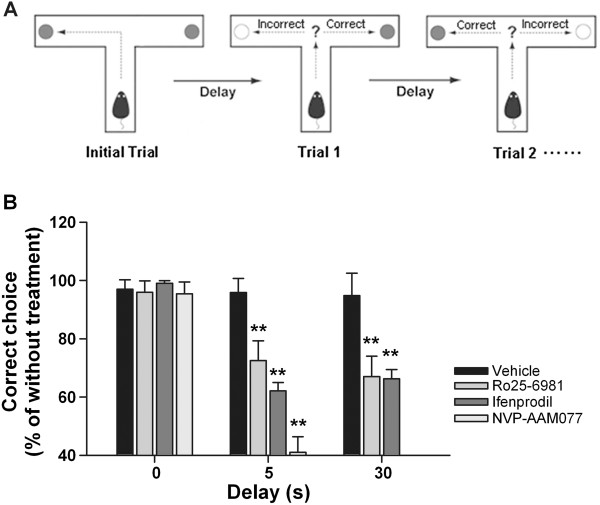
**Effect of intra-CA1 inhibition of NR2B-NMDARs on delayed-alternation T-maze task. (A)**Diagram showing the delayed-alternation performance in T-maze task. In the initial trial, both the left- and right-arm of the maze is baited and rats could get food by entering into either of them. From trial 1 on, rats have to avoid the arm visited in the previous trial and enter into the opposite arm in order to get reward. Inter-trial interval (delay period) is 0, 5, or 30 seconds. (**B**) Rats with intra-CA1 infusion of Ro25-6981, ifenprodil, or NVP-AAM077 made significantly more errors compared with the control rats in T-maze performance with 5-s or 30-s delay. **p<0.01 vs. the vehicle control, Mann-Whitney U-test.

To examine the roles of CA1 region NR2B- and NR2A-NMDARs in spatial working memory, we trained rats on a delayed alternation task in T-maze (DAT task). In order to perform the task correctly, rats had to remember during the delay which arm had been visited in the previous trial and select the opposite arm (Figure 1A). 0-s, 5-s, and 30-s delay was introduced between trials. When rats performed DAT task at ~80% correct on three consecutive days of testing, the NR2B antagonists, Ro25-6981 and ifenprodil, the NR2A preferring antagonist NVP-AAM077, or PBS were bilaterally infused into the CA1 region 15 min before the rats performed the task.

The rats treated with Ro25-6981 or ifenprodil, made significantly more errors than the controls in 5-s delay task and 30-s delay task respectively (Figure 1B; 5-s delay: *p* < 0.01 for Ro25-6981 *vs*. PBS, *p* < 0.01 for ifenfrodil *vs*. PBS, PBS: n = 7, Ro25-6981: n = 6, ifenprodil: n = 6; 30-s delay: *p* < 0.01 for Ro25-6981 *vs*. PBS, *p* < 0.01 for ifenfrodil *vs*. PBS, PBS: n = 7, Ro25-6981: n = 9, ifenprodil: n = 7;), whereas their performance showed no deficit compared with the control rats in 0-s delay task (Figure 1B; *p* > 0.05; PBS: n = 6, Ro25-6981: n = 6, ifenprodil: n = 6). NVP-AAM077-treated rats made dramatically more errors than the control rats in 5-s delay task (Figure 1B; *p* < 0.01, n = 6), and they could not perform the task when delay was extended to 30 s showing entered the same arm repeatedly (Data not shown). However, NVP-AAM077-treated rats made comparable errors with the control rats in 0-s delay task (Figure 1B; *p* > 0.05, n = 7).

**Figure 2:**
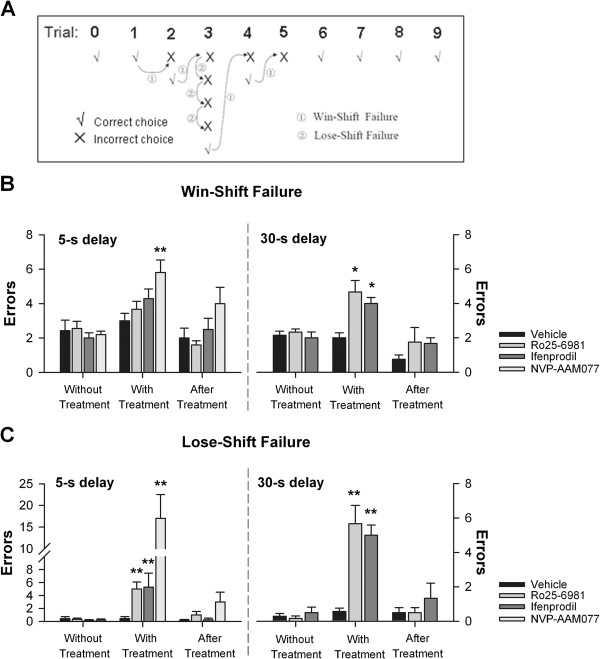
**Effect of intra-CA1 inhibition of NR2B-NMDARs on win-shift failure and lose-Shift failure in T-maze delayed-alternation task. (A)** Diagram showing the two types of performance errors in delayed-alternation T-maze task. *Win-shift* failure means that rats did not alter their choice after they made a correct selection in the previous trial, and *Lose-Shift* failure that the rats repeated an incorrect choice made in the previous trial. (**B**) Rats with intra-CA1 infusion of Ro25-6981, ifenprodil exhibited significantly more *Win-shift* failures relative to controls in 30-s delay (*right*), but not in 5-s delay (*left*), while NVP-AAM077-treated rats showed significantly more *Win-shift* failures in 5-s delay. *p<0.05, **p<0.01 *vs*. vehicle control, Mann-Whitney *U*-test. (**C**) Rats with intra-CA1 infusion of Ro25-6981, ifenprodil demonstrated significantly more *Lose-shift* failures in both 5-s (*left*) and 30-s delay (*right*), while NVP-AAM077-treated rats showed dramatically more *Lose-shift* failures in 5-s delay (*left*). **p<0.01 *vs*. the vehicle control, Mann-Whitney U-test.

In this task, we introduced a correction procedure in case rats made an error choice: the same arm was baited again, giving rats a chance to shift their selection. Rats received as many correction trials as necessary, i.e., the same arm was baited until they made a correct choice. As shown in Figure 2A, there were two types of performance errors: rats did not shift their choice after they selected a correct arm in the previous trial (*Win-shift failure*), or they repeated an incorrect choice that was made in the previous trial (*Lose-Shift failure*) (Figure 2A). *Win-shift failure* reflects a deficit in working memory, whereas *Lose-shift failure* a deficit in error-correction ability.

Interestingly, analysis of error types revealed that the rats treated with Ro25-6981 or ifenprodil showed no deficit in using *Win-shift* strategy (Figure 2B; *p* > 0.05) but an inability to use *Lose-shift* strategy in 5-s delay task (Figure 2C; *p* < 0.01 for Ro25-6981 vs. PBS; *p* < 0.01 for ifenprodil vs. PBS). When the delay was extended to 30 s, the rats treated with Ro25-6981 or ifenprodil made significantly more errors in using both *Win-shift* and *Lose-shift* strategies (Figure 2B-C; *p* < 0.01 for Ro25-6981 *vs*. PBS; *p* < 0.01 for ifenprodil *vs*. PBS). The NVP-AAM077-treated rats showed severely deficits in using *Win-shift* and *Lose-shift* strategies in 5-s delay task (Figure 2B-C, *p* < 0.01 for NVP-AAM077 *vs*. PBS). However, the rats treated with Ro25-6981, ifenprodil, or NVP-AAM077 performed equally well with the control rats when re-tested ~6 h post-treatment either in 5-s delay task or in 30-delay task (Figure 2B-C).

Our results indicated that inhibition of CA1 NR2B-NMDARs impaired an ability to use *Win-shift* strategy in a delay-dependent manner in DAT task.

Intra-CA1 inhibition of NR2B- NMDARs impairs spatial working memory in delayed matching-to-place water maze task with 10-min but not 30-s delay.

**Figure 3:**
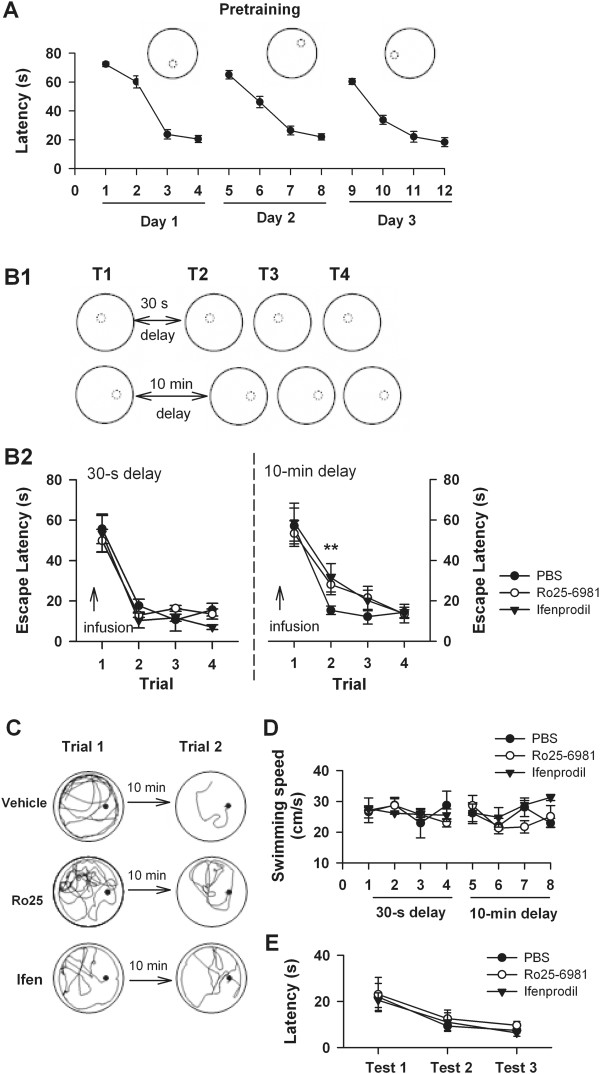
**Effects of intra-CA1 inhibition of NR2B -NMDARs on delayed matching-to-place water maze task. A**. Escape latency for each of the 4 trials per day of all rats during pretraining with changing platform position every day. Cutoff time was 90 s. Inset: the small dot-line circle indicated the position of invisible platform in the training session. **B**. Intra-CA1 inhibition of NR2B-NMDARs impairs the performance of delayed matching-to-place (DMP) water maze task. The DMP water maze protocol. Rats are given 4 trials per day (T1-T4) with the hidden platform staying in the same location. The platform moves location between days. The interval between trials 1 and 2 is 30 s or 10 min (B1). Rats treated with Ro25-6981 or ifenprodil showed deficit in the performance of DMP task with 10-min delay (*right*) but not 30-s delay (*left*). Arrows indicate that intra-CA1 infusion (B2). Representative swimming traces of each group of rats at DMP task with 10-min delay (B3). **C**. Intra-CA1 infusion of Ro25-6981 or ifenprodil had no effect on the motor ability and the motivation of rats. Rats with intra-CA1 inhibition of NR2B-NMDARs showed no difference b in swimming speed at the trial 2 of DMP task with 10-min delay (C1) and the visible platform task (C2).

To further test the role of CA1 region NR2B-NMDARs in spatial working memory, we trained rats on a delayed matching-to-place task in water maze (DMP task). In this task, the hidden platform was transferred to a novel location each day. In order to locate the platform in trial 2, rats had to learn this new location (in trial 1) and retained its spatial memory for short period of time (the interval between trial 1 and 2) [7,24,25]. During pre-training, the platform was transferred to a novel location each day and rats had to learn this new location and retained its spatial memory (Figure 3A). In the DMP task, 30-s or 10-min delay was introduced between trial 1 and 2, respectively (Figure 3B1). The escape latency in trial 2 reflects the performance of spatial working memory. Ro25-6981 or ifenprodil was infused into the CA1 region 15 min before rats performed the task. The rats treated with ifenprodil or Ro25-6981 exhibited no amnesia for the novel location of platform compared with the control rats in 30-s delay task (Figure 3B2; Trial 2: F (2, 19) = 0.39, *p* > 0.05; Trial 1: F (2, 19) = 0.03, *p* > 0.05; PBS: n = 7, Ro25-6981: n = 7, ifenprodil: n = 6). When the delay was extended to 10 min, the rats treated with ifenprodil or Ro25-6981 required significantly longer escape latency than the control rats (Figure 3B2; Trial 2, F (2, 23) = 6.13, *p* < 0.05 for whole effect; *p* < 0.05 for Ro25-6981 *vs*. PBS; *p* < 0.05 for ifenprodil *vs*. PBS; PBS: n = 8, Ro25-6981: n = 8, ifenprodil: n = 8). An ANOVA revealed no group effect on escape latency in trial 1 (Figure 3B2; Trial 1, F (2, 23) = 0.02, *p* > 0.05). The rats treated with Ro25-6981 or ifenprodil showed no deficit in both swimming speed of the trial 2 (Figure 3C1; *p* > 0.05) and escape latency of the visible platform test (Figure 3C2; *p* > 0.05). These data suggested that the longer escape latency in trial 2 induced by intra-CA1 infusion of Ro25-6981 or ifenprodil was not due to impairment in visual discrimination, swimming ability or motivation.

**Figure 4:**
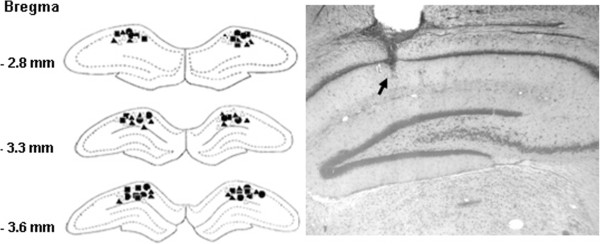
**The histological confirmation of the infusion sites of the drugs in the CA1 region*.*** Reconstructions of the infusion sites (*left*) and representative coronal section showing the infusion site (*right*). PBS: filled circles; NVP-AAM077: filled squares; Ro25-6981: open triangles; ifenprodil: filled triangles.

These results indicated that intra-CA1 inhibition of NR2B-NMDARs delay-dependently impaired spatial working memory in DMP task Figure 4.

## Discussion

The present study aim to evaluate the role of CA1 NR2B-NMDARs in spatial working memory in a time window involving a range of short-to-long delays in two behavioral paradigms. We investigate the effect of intra-CA1 pharmacological inhibition of NR2B-NMDARs on spatial working memory while the well-trained rats perform a DAT task or a DMP task with the different delay periods. Intra-CA1 infusions of NR2B-NMDAR selective antagonists severely impair the spatial working memory in both tasks with long delays (30 s and 10 min, respectively) but not with short delays (5 s and 30 s, respectively). However, intra-CA1 inhibition of NR2A-NMDAR preferring antagonist severely impair spatial working memory even in short-delay DAT task.

NMDAR have been proposed to be essential for spatial working memory in a delay-dependent manner in the CA1 region. For example, pharmacological blockade of NMDAR in area CA1 produced a delay-dependent deficit in spatial working memory in a DAT task [18,20] and a delay-dependent deficit in 8-arm radial maze [19]. Similar delay-dependent deficit in spatial working memory was also observed in a DMP task in water-maze following intra-CA1 blockade of NMDAR [7]. The importance of NMDAR subunits NR2A and NR2B for spatial working memory has been reported in genetic manipulation mice in the CA1 region [22,23], although these studies did not clarify whether or not the requirements of NR2A and NR2B subunits in spatial working memory are delay-dependent. Our main finding is that NR2B-NMDARs, but not NR2A-NMDARs, are delay-dependently required for the spatial working memory in the CA1 region.

In the present study, we demonstrate that NR2B-NMDARs contribute to spatial working memory in DAT task and DMP task with long delays, but not short delays. Definitions of long delays or short delays are relative to the time finishing each trial in the tasks. In our study, the well-trained rats finish each trial within few seconds (~ 5 s) in T-maze task, while the rats need to take several ten seconds (~ 60 s) to locate the hidden platform at a “novel” place in DMP water maze task. Our data suggest that the time scale of the long delay, by which a spatial working memory deficit is induced in NR2B-NMDAR inhibited rats, may vary across tasks. For example, a delay of 30 s is sufficient to see a deficit in NR2B-NMDAR antagonist treated rats in DAT task (Figure 2) but not in DMP task, where a deficit is observed when a delay of 10 min is imposed (Figure 3). This variation is mainly due to the different time scale of memory formation. For example, spatial memory about previous visited arm is formed within several seconds in T-maze task, while memory about the spatial information of the platform is acquired in about one minute in DMP water maze task. Consistently, Caramanos and Shapiro (1994) found that the sensitivity of a delay-imposed radial-maze performance to AP5 depends on environmental familiarity [26].

The delay-dependent impairment seen with intra-CA1 inhibition of NR2B- but not NR2A-NMDARs in T-maze task is the most novel finding of this experiment. The DAT task is a classic spatial working memory task in rodent and requires the prefrontal-hippocampus neural circuits [27]. In this task, the rats are required to remember the spatial location of a reward and the relevance of this spatial information is short-maintained (delay duration ≤ 60 s) [28,29]. Errors were divided into two types: *Win-shift failure* and *Lose-Shift failur* (Figure 2A). Working memory has been proposed to be a multi-component system, involving short-term storage of information and executive functions [30]. *Win-shift failure* imply defects in short-term storage of spatial information (working memory ability), and *Lose-shift failure* reflect an error-correction ability indicating perseverative behaviour or disability of executive functions [31,32]. Interestingly, we find that NR2B-NMDARs are important for working memory ability in the DAT task with long delay (30 s) but not short delay (5 s) (Figure 2B), whereas are critical for error-correction ability even in short-delay imposed DAT task (Figure 2C), while NR2A-NMDARs are critical for working memory ability and error-correction ability in short-delay DAT task. Both working memory ability and error-correction ability is sensitive to the function of hippocampus with lesion to hippocampus increasing these two types of errors [27,33]. Our finding of increased *Lose-shift failure* after hippocampal NR2A- or NR2B-NMDAR inhibition is in line with the previous study using NMDAR non-selective antagonist [34]. One argue is that deficit in spatial working memory may due to inability to use the rule of T-maze task. However, our data show that inhibition of NR2B- or NR2A-NMDARs does not affect the ability to utilize the rule of the T-maze task, as such that the rats with inhibition of either NR2B- or NR2A-NMDARs keeps intact working memory in 0-s delay task (Figure 1B). Furthermore, neither NR2B- nor NR2A-NMDAR antagonist induce sensory or motor disturbances to account for deficits in working memory ability and error-correction ability.

The delay-dependent impairment induced by intra-CA1 inhibition of NR2B-NMDARs in DMP task in water maze is consistent with the previous study with intra-CA1 infusion of NMDAR non-selective antagonist APV [7]. The DMP water maze task is a repeated one-trial memory task that is very sensitive to hippocampal dysfunction and allows the training across successive days with a variable delay [7]. In this task, the hidden platform is always in a “novel” location on the first trial of each day, and remains in a fixed location for the subsequent trials of that day (i.e., trials 2, 3 and 4). In order to locate the platform in trial 2, rats have to learn this new location at trial 1 and retain its spatial information for short period of time (interval between trial 1 and 2). Trial 2 serves as a memory retrieval trial to investigate the retention of spatial information acquired in trial 1. Trials 3 and 4 serve only to sustain the win-stay strategy of swimming back to the place in the pool where escape has been possible most recently. Therefore, the DMP water maze task has the most important feature of working memory that the acquired spatial information is only useful for a period. In the present study, NR2B-NMDAR antagonist does not disrupt the visual discrimination and did not have major effects on swimming speed or motivation to perform the DMP task (Figure 3C1-C2). The acquisition of spatial memory is unaffected as such no impact on the memory in 30-s delay task with pre-trial 1 inhibition of NR2B-NMDARs (Figure 3B2). A retrieval deficit is also ruled out because pre-test inhibition of NR2B-NMDARs has no effect on spatial reference memory in the probe test of removing platform (Data not shown). Therefore, NR2B-NMDAR antagonist induced deficit in spatial working memory may result from the impairment of the rapid consolidation of spatial information in DMP task.

In addition, delay-dependent requirement of hippicampal NR2B-NMDARs is not limited to spatial working memory. The requirement of NR2B-NMDARs for associate memory, e.g. fear memory, has been proposed to be conditioning-strength dependent in some brain regions, including the CA1 region [15,16,35]. Pre-training inhibition of NR2B-NMDARs impairs five Cs-Us pairings induced fear memory, with no impact on one Cs-Us pairing induced fear memory. Meanwhile, the contribution of NR2B-NMDARs to synaptic plasticity (Long-term potentiation, LTP) depends on induction protocol in adult hippocampus. Pharmacological inhibition of NR2B-NMDARs suppresses LTP induced by spike-timing protocol, with no impact on LTP induced by pairing protocol [16]. Under this condition, it is hard to distinguish whether the conditioning strengh or repeptive stimulations activate NR2B-NMDARs. However, the different pattern in kinetics of intracellular Ca^2+^ signals induced by these two protocols may most likely account for the protocol-dependent involvement of NR2B-NMDARs in hippocampal LTP [16]. Recently, several important studies reveal an important role of Ca^2+^ influx through NR2B (not NR2A)-NMDARs in memory formation [36,37], especially, hippocampus-dependent short-term memory formation [37], suggesting that Ca^2+^ influx through NR2B- or NR2A-NMDARs plays the different roles in memory formation. Based on these findings, we propose that the rapid memory formation, which is required by short-delay working memory tasks, only requirs NR2A-NMDARs but not NR2B-NMDARs, while NR2B-NMDARs contribute to short-term memory formation in long-delay working memory tasks, although the difference of kinetics of Ca2+ influx induced by short-delay tasks and long-delay tasks is unknown.

Working memory is a dynamic and goal-directed active representation of information over a short time and requires the prefrontal-hippocampus neural circuits [38,39]. It is well established that the critical role of prefrontal cortical NR2B-NMDARs in working memory, such as persistent neuronal firing required by working memory is highly dependent on NR2B-NMDARs in the prefrontal cortex [40,41]. The fact that intra-CA1 inhibition of NR2B-NMDARs impairs spatial working memory in a delay-dependent manner raises an important question: does spatial working memory require NR2B-NMDAR mediated synaptic plasticity in the CA1? The answer seems to be yes.

In conclusion, our results provide the first evidence that NR2B-NMDARs are required for the spatial working memory in a delay-dependent manner in the CA1 region. It means that NR2B-NMDARs are critical for the spatial working memory in the tasks with long delay (not short delay).

## Materials and methods

### Subjects

Sprague-Dawley rats (male, 200-220 g, 8-10 weeks old) were purchased from SLACCAS (Shanghai, China). Rats were housed in plastic cages (1-2 per cage) and maintained on a 12 h light/12 h dark cycle. Food and water were available *ad libitum*. All experimental procedures were approved by the Ethical Committee of Rat Experiments at the Fudan University Institute of Neurobiology (Shanghai, China) and were in accordance with the National Institutes of Health *Guide for the Care and Use of Experimental Rats* (1996).

### Chemicals

Two selective NR2B-NMDAR antagonists: the non-competitive NR2B antagonist ifenprodil tartrate salt (Sigma-Aldrich, St. Louis, MO) and its derivative Ro25-6981 hydrochloride (Tocris, UK) were used. We used the 5.0 μg dose of Ro25-6981 (in 1.0 μl, 0.01 M phosphate-buffered saline, PBS) and the 2.0 μg dose of ifenprodil (in 1.0 μl PBS). We used NR2A-NMDAR preferring antagonist NVP-AAM077, a generous gift from Novartis Pharma (Basel, Switzerland), we employed the 0.12 μg dose of NVP-AAM077 in 1.0 μl PBS [15,16].

### Surgery and drug administration

Rats were anesthetized with pentobarbital sodium (40 mg/kg, i.p.). Guide cannulae (23-guage) were bilaterally implanted into the dorsal hippocampus (3.2~3.3 mm posterior to Bregma, 1.5~1.8 mm lateral to the midline, and 1.7 mm beneath the surface of the skull). Dummy cannulae were inserted into the guide cannulae to prevent clogging and reduce risk of infection. Rats were given at least 5 days to recover before behavioral training.

For drug infusion, the dummy cannulae were removed and injection needles (30-guage) were inserted into the guide cannulae. The tips of the injection needle were 1.5 mm lower than those of the guide cannulae, i.e., at a location 3.2 mm ventral to the skull surface. Drug solutions or PBS (1.0 μl each side) were bilaterally infused into the CA1 region, at the rate of 0.5 μl /min using a pump. The injection needles were left in place for an additional 2 min after infusion. Experiments were carried out in a double-blind way.

### Behavioral procedure

#### Delayed alternation T-maze task

T-maze consists of a stem (50×10cm) and two arms (40×10cm) with walls (20 cm high). A sliding door separated the first 15 cm of the stem as a starting compartment. Rats learned to visit the two arms alternatively in order to obtain a food reward (Figure 1A).

Rats were trained on a delayed-alternation task in T-maze (DAT task) with minor modifications [42]. Rats were subject to restrict diet and maintain at approximately 85% of their original weight for 1 week. They were habituated to a T-maze until they voluntarily ate a piece of peanut placed at the end of each arm. Each daily session consisted of 10 trials, including one initial trial and 9 formal trials. Each trial began by removing the sliding door and allowing rats to explore the maze. In the initial trial, the two arms were both baited and rats got reward by visiting either of them. From formal trial 1 through 9, rats had to avoid the arm once visited in a previous trial and select the opposite arm to get reward. Rats were guided back into the starting compartment after each trial was completed and the sliding door was closed. The next trial began after an interval of 0, 5 or 30 seconds (delay). In the experiment, we defined the delay as the interval between the sliding door closing and opening, e.g. 0-s delay is that the sliding door opened immediately after closed. T-maze was wiped with alcohol to remove any olfactory clues between trials. Once rats’ performance was stable at ~ 80% correct on three consecutive days of testing, intra-CA1 infusions were initiated.

In order to perform the task correctly, rats had to remember during the delay which arm had been visited in the previous trial and select the opposite arm. It is a delayed nonmatching-to-place (DNMP) task. If rats selected the un-baited arm, a self-correction procedure was introduced by keeping the baited one still baited until it was visited, giving the rats a chance to shift their choice. The errors were divided into two types: a *win-shift failure* was defined as the rat entering the arm that was the correct choice in the previous trial; a lose-shift failure was defined as the rat continuing to enter an arm that was the wrong choice in the previous trial.

#### Delayed matching-to-place water maze task

The water maze consisted of a black round tank (150 cm in diameter and 54 cm in height) filled to a depth of 38 cm with water (temperature 26 ± 2°C). The water was made opaque so that the submerged platform (9.0 cm in diameter, 2.0 cm below the water surface) was invisible.

##### Delayed matching-to-place water maze task

Rats were first trained to find the platform at the fixed position in one day to ensure that they had acquired basic procedural components of the task as well as a correct representation of the environment. The fixed-platform training procedure refers to our previous work [43]. Then rats were began 3-day pre-training with changing platform position every day (4 trials per day, inter-trial interval for trial 1-4: 30 s; Figure 1A). Trials began at one of four starting points (i.e. N, S, E or W), in a pseudorandom sequence, with the rats at and facing the sidewall. The rat was allowed to search for the submerged platform for 90 s. If successful in locating the platform within 90 s, the rat was allowed to stay on the platform for 30 s; if not, it was guided to the platform and allowed to stay there for 30 s. Thereafter, the rat was returned to a holding cage to stay 60 s before the next trial began. Following the 3-day pre-training, rats were tested on delayed matching-to-place (DMP) water maze task with one session each day. Each session contains four trials (inter-trial interval for trial 2-4: 15 s), and different delays (30 s or 10 min) were introduced between trial 1 and 2 (both ITIs are not including a 30-s period spent on the platform). Therefore, trial 2 reflects memory of information acquired in trial 1 and was given particular weight during data analysis. PBS, ifenprodil, or Ro25-6981 was infused into CA1 region 10 min before the trial 1 was began.

##### Visible platform testing

Immediately after DMP testing, the rats were tested in a visible platform version of the water maze as described in our previous work [43]. Briefly, the platform was raised to above the water surface and was covered with white gauze to be highly visible. Each rat was placed on the visible platform for 30 s prior to testing. The starting position for any given rat from the groups was selected randomly, but once selected it was fixed for that rat, whereas the visible platform was randomly placed among the four quadrants. The rat was allowed to locate the visible platform for 60 s in each trial. If successful in finding the platform, the rat was returned immediately to a holding cage; if not, the rat was removed from water and returned to a holding cage. The next trial began after an inter-trial interval of 60 s. Three trials were conducted for each rat.

Navigation of each rat in the water maze was monitored using a video camera, a tracking system and tracking software (San Diego Instruments, USA). Escape latency, distance swam and swimming speed was recorded for subsequent analysis.

#### Histology

Rats were anesthetized with an overdose of pentobarbital sodium (50 mg/kg, i.p.) and perfused transcardially with saline, followed by 4% (vol/vol) formaldehyde solution. Rat brains were removed, placed into 30% (wt/vol) sucrose solution, and subsequently cut into 40~50 μm sections with a cryostat (Leica CM900, Germany). Brain sections were mounted on gelatin-subbed glass slides and stained with neural red (1% in ddH2O). Images were taken using a light microscope (Leica DMRXA Q5001W) equipped with a CCD camera.

#### Data analysis

Data in the text and figures are expressed as means ± SEM. For DMP water maze task, data between groups were statistically campared using one-way Analysis of Variance (ANOVA) with planned comparisons as *post hoc* analysis. For DAT task, data between groups were statistically compared using Mann-Whitney *U*-test. *p*<0.05 was considered significant. ANOVA was conducted using SigmaStat (Systat Soft Inc., USA).

## Competing interests

The authors declare that they have no competing interests.

## Authors’ contributions

ZXH designed experiment, analyzed data, wrote manuscript. LSS and ZXH carried out T-maze task experiment; YF and ZXH carried out water maze task experiment. LBM and ZM conceived of the study. All authors read and approved the final manuscript.
